# Drug Resistance Characteristics of *Mycobacterium tuberculosis* Isolates From Patients With Tuberculosis to 12 Antituberculous Drugs in China

**DOI:** 10.3389/fcimb.2019.00345

**Published:** 2019-11-05

**Authors:** Xiaocui Wu, Jinghui Yang, Guangkun Tan, Haican Liu, Yin Liu, Yinjuan Guo, Rongliang Gao, Baoshan Wan, Fangyou Yu

**Affiliations:** ^1^Department of Clinical Laboratory, Shanghai Pulmonary Hospital, School of Medicine, Tongji University, Shanghai, China; ^2^Shanghai Key Laboratory of Tuberculosis, Shanghai Pulmonary Hospital, Tongji University School of Medicine, Shanghai, China; ^3^Department of Clinical Laboratory, Shanghai University of Traditional Chinese Medical Attached Shuguang Hospital, Shanghai, China; ^4^State Key Laboratory of Infectious Disease Prevention and Control, Chinese Center for Disease Control and Prevention, Beijing, China

**Keywords:** *Mycobacterium tuberculosis*, drug resistance characteristics, drug susceptibility testing, MDR-TB, XDR-TB

## Abstract

**Objective:** To investigate the drug resistance characteristics of *Mycobacterium tuberculosis* (MTB) isolates from patients with tuberculosis to 12 antituberculous drugs in China.

**Methods:** All clinical isolates of MTB were isolated from patients with tuberculosis in Shanghai Pulmonary Hospital (SPH) during the period from January 1st to December 31th, 2018. Drug susceptibility testing (DST) was performed in micro plates with 12 antituberculous drugs in accordance with relevant guideline. Demographic information, including sex, age, and treatment history was recorded.

**Results:** A total of 1,950 MTB isolates were included in this retrospective study which were isolated from 1,950 patients from 29 regions in China. One thousand six hundred and forty-four were initial treated and 306 were re-treated in the hospital. Two hundred and eight (10.67%, 208/1,950) cases were diagnosed as multidrug-resistant tuberculosis (MDR-TB), from which 74 (4.50%, 74/1,644) cases were initial treated, and the remaining (43.79%, 134/306) were re-treated cases. Besides, the percentage of extensively drug-resistant tuberculosis (XDR-TB) varied in such 3 different groups: 1.64% (32/1,950) in total cases, 0.30% (5/1,644) in initial treated cases and 8.82% (27/306) in re-treated cases. The total resistance rates were as follows: isoniazid (361, 18.51%), streptomycin (302, 15.49%), rifampin (241, 12.36%), ofloxacin (239, 12.26%), moxifloxacin (232, 11.90%), rifabutin (195, 10.00%), ethambutol (100, 5.13%), cycloserine (55, 2.82%), kanamycin (48, 2.46%), ethionamide (40, 2.05%), amikacin (39, 2.00%), and aminosalicylic acid (21, 1.08%). Rates of resistance to any drug in re-treated cases were significantly higher than in initial treated cases. The drug resistance rates of the 12 drugs were higher in males than in females. Patients older than 60 years had significantly lower percentages of MDR/XDR-TB (7.11 and 0.65%) than in younger age groups. The proportion of re-treated cases in Shanghai (11.38%, 88/773) was lower than that in other regions. Meanwhile, the percentages of MDR/XDR-TB in Shanghai (4.79 and 0.65%) were significantly lower than in other regions.

**Conclusions:** In this study, we found higher proportion of MDR/XDR-TB among re-treated cases than initial treated cases in China and the drug resistance rate of tuberculosis varied with age, sex, and region, indicating that standardized anti-tuberculosis treatment can reduce the incidence of drug-resistant tuberculosis and the recurrence of tuberculosis.

## Introduction

Drug-resistant tuberculosis (TB) is a global issue and an important public health concern. The amount of rifampin-resistance tuberculosis (RR-TB) was estimated to be 201.8 million worldwide in 2017, and 82% of such cases were multidrug-resistant TB (MDR-TB, resistance to at least isoniazid and rifampin) (World Health Organization, 2018). Globally, 3.5% of initial treated TB and 18% of re-treated TB had MDR/RR-TB. Among cases of MDR-TB in 2017, 8.5% were estimated to have extensively drug-resistant TB (XDR-TB, resistance to isoniazid, rifampin, one fluoroquinolone, and one second-line injectable drug) (World Health Organization, 2018). The period of treatment for MDR/RR-TB is longer than that for drug sensitive TB, accompanied by more expensive toxic drugs. The latest data of therapeutic outcome showed treatment success rates of 82% for TB, 55% for MDR/RR-TB and 34% for XDR-TB (World Health Organization, 2018).

The countries with the largest numbers of MDR/RR-TB cases (almost half of the world's cases) were India (24%), China (13%), and the Russian Federation (10%) (World Health Organization, 2018). In China, there were an estimated 7.1% of initial treated TB cases with MDR/RR-TB and an estimated 14% of re-treated TB cases with MDR/RR-TB (World Health Organization, 2018). The prevalence of MDR-TB varied from region to region, 14.3% in Guizhou (Lan et al., 2019), 8.0% in Hangzhou (Li et al., 2018), 13.3% in Sichuan (Zhou et al., 2018), 13.2% in Hebei (Li et al., 2016), 9% in Beijing (Liu et al., 2018), and 5% in Shanghai (Yang et al., 2017).

Recently, many studies had reported drug resistance characteristics of MTB clinical isolates in different regions of China, but there were shortcomings in covering a small number of anti-TB drugs or a single area. The aim of this study is to update drug resistance characteristics of MTB clinical isolates to four first-line antituberculous drugs including ethambutol, isoniazid, rifampin and streptomycin as well as eight second-line drugs including amikacin, cycloserine, ethionamide, kanamycin, moxifloxacin, ofloxacin, aminosalicylic acid, and rifabutin in China.

## Materials and Methods

### Study Population

This study was conducted in Shanghai Pulmonary Hospital (SPH), affiliated to Tongji University School of Medicine. SPH is a tertiary-care hospital and one of the national designated tuberculosis hospitals in China. Laboratory examinations were performed in the Clinical laboratory, an ISO 15189 accredited laboratory specialized in MTB detection.

All strains were isolated from culture-positive MTB cases that were diagnosed and treated in SPH in the whole year of 2018. For patients who were hospitalized many times, only the first batch of isolates acquired in this hospital were included to this study. The TB diagnostic criteria were based on the Chinese Pulmonary Tuberculosis Diagnostic Criteria (WS 288–2017) and the corresponding WHO guidelines. Demographic information, including sex, age, and treatment history was recorded for further analysis.

### MTB Culture and Identification

Clinical specimens, including sputum, bronchoalveolar lavage fluid, fine-needle aspiration (FNA) tissues, pleural fluid and other body fluids were collected from patients with suspected TB and subjected to cultivation with Bactec MGIT 960 instrument (Becton Dickinson, Cockeysville, MD, USA) in accordance with relevant guidelines, then positive cultures were switched to Lowenstein-Jensen medium (Baso, Zhuhai, China). All the MTB isolates were validated by both the growth test on p-nitrobenzoic acid containing medium (Baso, Zhuhai, China) and MBP 64 antigen detection kit (Genesis, Hangzhou, China). Nontuberculosis mycobacteria (NTM) were excluded.

### Drug Susceptibility Testing (DST)

The drug susceptibility testing (DST) was performed using Myco TB system (MYCOTB; Trek Diagnostic Systems, ThermoFisher Scientific Inc., USA). All steps were performed by trained and specialized persons in a biosafety cabinet in accordance with relevant guidelines. In brief, colonies were sweeped from the agar plates and suspended in sterile saline containing 0.2% tween and glass beads. After vortexing for at least 30 s to break up organism clumps, the bacterial suspension was sit for 15 min to allow any remaining clumps to settle to the precipitation, and the supernatant was adjusted to make a suspension with a turbidity of 0.5 McFarland standard using a nephelometer. One hundred microliter suspension was added to Middlebrook 7H9 broth containing oleic acid-albumin-dextrose-catalase (OADC) and then vortexed for 30 s. One hundred microliter of the subsequent suspension was added to each well of Myco TB plate. Myco TB plates were then covered with an adhesive seal and wiped with a disinfectant. Plates were incubated at 37°C for 10 days and checked for growth at 7–10 days. If growth was insufficient after 10 days, reincubate the plate up to an additional 11 days. All Sensititre® plates include positive control wells. All results are invalid unless there is distinct growth in the positive control well. The reference strain H37Rv should be used for quality control once a month or every new batch of susceptibility kit. Results can be read using the Vizion® System. Growth appears as turbidity or as a deposit of cells at the bottom of a well.

The MIC value is the lowest drug concentration that significantly inhibits visible growth. The critical concentration of *Mycobacterium tuberculosis* drug sensitivity test is based on CLSI M24-A2 and FDA-approved standards for drug susceptibility testing. Among them, the critical concentration of cycloserine is derived from the FDA-approved standards (https://www.accessdata.fda.gov/spl/data/a0f29de7-d7cd-45a0-915a-eae2562edaf8/a0f29de7-d7cd-45a0-915a-eae2562edaf8.xml), and the rest are from CLSI M24-A2 (Woods et al., 2011; Zhou et al., 2018).

The drug concentration range and the critical concentration of Myco TB are shown in Table 1. The MIC value of a strain resistant to a certain drug is higher than the critical concentration, and if the MIC value is less than or equal to the critical concentration, then such strain is sensitive to the drug.

**Table 1:** The drug concentration ranges and the critical concentrations of Myco TB.

**Drug**	**Concentration range (μg/mL)**	**Critical concentration (μg/mL)**
Amikacin	0.12~16	4
Cycloserine	2~256	25
Ethambutol	0.5~32	5
Ethionamide	0.3~40	5
Isoniazid	0.03~4	0.2
Kanamycin	0.6~40	5
Moxifloxacin	0.06~8	0.5
Ofloxacin	0.25~32	2
Aminosalicylic acid	0.5~64	2
Rifabutin	0.12~16	0.5
Rifampin	0.12~16	1
Streptomycin	0.25~32	2

### Statistical Analysis

All data were entered into Microsoft Office Excel (Microsoft). Statistical analysis was performed with the WPS software (Kingsoft, China). This map of China showing the distribution of 1,950 isolates included in this study was drawn in R language.

## Results

### Distribution of Isolates

**Figure 1:**
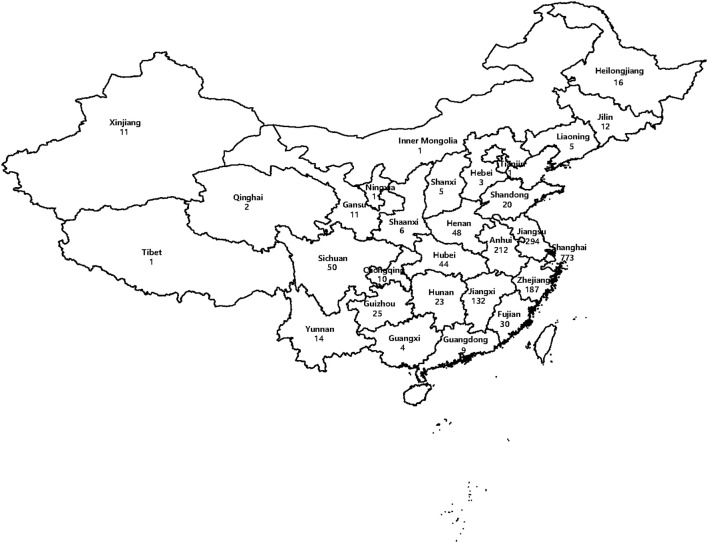
Map of China showing the distribution of 1,950 isolates included in this study (the numbers indicate the absolute number of isolates every region).

One thousand nine hundred and fifty MTB isolates were collected from patients who were diagnosed as TB from 29 provinces, autonomous regions, and municipalities in China. For all the 1,950 isolates, 773 were from Shanghai, 294 from Jiangsu Province, 212 from Anhui Province, 187 from Zhejiang Province, 132 from Jiangxi Province, 50 from Sichuan Province, and 302 from other districts (Figure 1). According to the regional distribution of China, these provinces, autonomous regions and municipalities are divided into eastern regions, central regions, and western regions. The eastern regions include Shanghai, Jiangsu Province, Zhejiang Province, Fujian Province, Shandong Province, Guangdong Province, Liaoning Province, Heilongjiang Province, Jilin Province, Hebei Province, and Tianjin. The central regions include Anhui Province, Jiangxi Province, Henan Province, Hubei Province, Hunan Province and Shanxi Province. The western regions include Sichuan Province, Guizhou Province, Yunnan Province, Gansu Province, Xinjiang Uygur Autonomous Region, Chongqing, Shaanxi Province, Guangxi Province, Qinghai Province, Inner Mongolia Autonomous Region, Tibet Autonomous Region and Ningxia Hui Autonomous Region. Among 1,950 isolates, the majority from eastern regions (1,350, 69.23%), followed from central regions (464, 23.80%) and western regions (136, 6.97%). Figure 1 shows the geographical distribution of these isolates.

### Demographic Characteristics of the Included Patients

The demographic characteristics of the included patients are shown in Table 2. Among these patients, 1,303 (1,303/1,950, 66.82%) were males whose ages ranged from 2 to 95 years old (mean: 42.5 years old). There was 1,644 initial treated TB cases and 306 re-treated TB cases based on treatment history. Most of the patients (1,350, 69.23%) came from the eastern region. The types of specimens included sputum (1,021, 52.4%), bronchoalveolar lavage fluid (707, 36.3%), FNA tissues (82, 4.2%), pleural fluid (67, 3.4%), and other body fluids (73, 3.7%).

**Table 2:** Demographic characteristics and specimen types in this study (*n* = 1,950).

**Characteristics**		**No**
Sex	Male	1,303
	Female	647
Age	≤ 20	151
	21–40	790
	41–60	545
	>60	464
Treatment history	Initial treated TB	1,644
	Re-treated TB	306
Region	Eastern Regions	1,350
	Central regions	464
	Western Regions	136
Clinical specimen types	Sputum	1,021
	Bronchoalveolar lavage fluid	707
	FNA tissues	82
	Pleural fluid	67
	Other body fluids^a^	73

a *Other body fluids include cerebrospinal fluid, peritoneal fluid, pericardial fluid, etc*.

### Drug-Resistance Patterns of MTB Isolates

**Figure 2:**
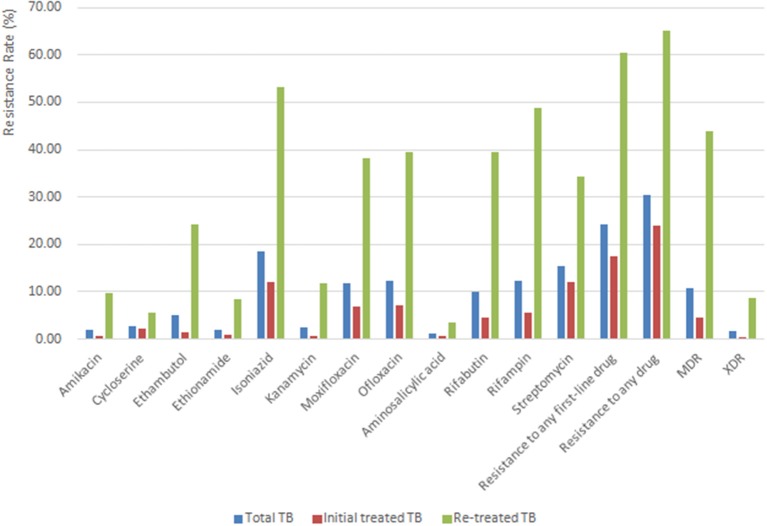
The drug resistance profile of all 1,950 *M. tuberculosis* isolates.

Figure 2 shows the drug resistance profile of all 1,950 *M. tuberculosis* isolates. Of the 1,950 isolates, 30.31% (591/1,950) were resistant to at least one drug, and 24.26% (473/1,950) were resistant to at least one first-line drug. The total resistance rates were as follows: isoniazid (361, 18.51%), streptomycin (302, 15.49%), rifampin (241, 12.36%), ofloxacin (239, 12.26%), moxifloxacin (232, 11.90%), rifabutin (195, 10.00%), ethambutol (100, 5.13%), cycloserine (55, 2.82%), kanamycin (48, 2.46%), ethionamide (40, 2.05%), amikacin (39, 2.00%), and aminosalicylic acid (21, 1.08%). 10.67% (208/1,950) of TB cases were diagnosed as MDR-TB, 4.50% (74/1,644) among initial treated TB cases and 43.79% (134/306) among re-treated TB cases. The percentage of XDR-TB varied in different groups of people as follows: 1.64% (32/1,950) in total TB cases, 0.30% (5/1,644) in initial treated TB cases and 8.82% (27/306) in re-treated TB cases. Rates of resistance to any drug in re-treated TB cases were significantly higher than those in initial treated TB cases (Figure 2).

Out of the 208 MDR isolates tested, 169 (81.25%) were resistant to rifabutin, 135 (64.90%) to streptomycin, 114 (54.81%) to ofloxacin, 110 (52.88%) to moxifloxacin, 85 (40.87%) to ethambutol, 38 (18.27%) to kanamycin, 33 (15.87%) to ethionamide, 32 (15.38%) to amikacin, 20 (9.62%) to cycloserine and 14 (6.73%) to aminosalicylic acid.

Among 241 RR-TB isolates, 208 (86.31%) isolates were resistant to isoniazid and 193 (80.08%) were resistant to rifabutin. Nearly 80% of kanamycin-resistant isolates (79.17%, 38/48) were also amikacin-resistant isolates. The majority of the ofloxacin-resistant isolates (94.98%, 227/239) were also resistant to moxifloxacin.

### Drug Resistance Profile in Different Genders and Age Groups

As shown in Table 3, the drug resistance rates of the 12 drugs were higher in males than in females. The percentages of MDR/XDR-TB in males (11.67 and 2.00%) were higher than in females (8.66 and 0.93%). Patients older than 60 years had significantly lower percentages of MDR/XDR-TB (7.11 and 0.65%) than in younger age groups.

**Table 3:** The drug resistance profile in different genders and age groups.

**Drug**	**Sex**						**Age**											
	**Male (*****N*** **= 1,303)**			**Female (*****N*** **= 647)**			**≤20 (*****N*** **= 151)**			**21-40 (*****N*** **= 790)**			**41-60 (*****N*** **= 545)**			**>60 (*****N*** **= 464)**		
	**Initial treated TB (*N* = 1,068)**	**Re-treated TB (*N* = 235)**	**Total TB**	**Initial treated TB (*N* = 576)**	**Re-treated TB (*N* = 71)**	**Total TB**	**Initial treated TB (*N* = 138)**	**Re-treated TB (*N* = 13)**	**Total TB**	**Initial treated TB (*N* = 693)**	**Re-treated TB (*N* = 97)**	**Total TB**	**Initial treated TB (*N* = 418)**	**Re-treated TB (*N* = 127)**	**Total TB**	**Initial treated TB (*N* = 395)**	**Re-treated TB (*N* = 69)**	**Total TB**
Amikacin	5 (0.47%)	23 (9.79%)	28 (2.15%)	4 (0.69%)	7 (9.86%)	11 (1.70%)	3 (2.17%)	1 (7.69%)	4 (2.65%)	2 (0.29%)	10 (10.31%)	12 (1.52%)	3 (0.72%)	17 (13.39%)	20 (3.67%)	1 (0.25%)	2 (2.90%)	3 (0.65%)
Cycloserine	28 (2.62%)	14 (5.96%)	42 (3.22%)	10 (1.74%)	3 (4.23%)	13 (2.01%)	6 (4.35%)	1 (7.69%)	7 (4.64%)	16 (2.31%)	6 (6.19%)	22 (2.78%)	9 (2.15%)	9 (7.09%)	18 (3.30%)	7 (1.77%)	1 (1.45%)	8 (1.72%)
Ethambutol	18 (1.69%)	58 (24.68%)	76 (5.83%)	8 (1.39%)	16 (22.54%)	24 (3.71%)	6 (4.35%)	4 (30.77%)	10 (6.62%)	9 (1.30%)	29 (29.90%)	38 (4.81%)	8 (1.91%)	33 (25.98%)	41 (7.52%)	3 (0.76%)	8 (11.59%)	11 (2.37%)
Ethionamide	10 (0.94%)	22 (9.36%)	32 (2.46%)	4 (0.69%)	4 (5.63%)	8 (1.24%)	2 (1.45%)	1 (7.69%)	3 (1.99%)	6 (0.87%)	10 (10.31%)	16 (2.03%)	5 (1.20%)	12 (9.45%)	17 (3.12%)	1 (0.25%)	3 (4.35%)	4 (0.86%)
Isoniazid	138 (12.92%)	121 (51.49%)	259 (19.88%)	60 (10.42%)	42 (59.15%)	102 (15.77%)	16 (11.59%)	9 (69.23%)	25 (16.56%)	83 (11.98%)	60 (61.86%)	143 (18.10%)	51 (12.20%)	71 (55.91%)	122 (22.39%)	48 (12.15%)	33 (47.83%)	71 (15.30%)
Kanamycin	8 (0.75%)	28 (11.91%)	36 (2.76%)	4 (0.69%)	8 (11.27%)	12 (1.85%)	5 (3.62%)	1 (7.69%)	6 (3.97%)	1 (0.14%)	13 (13.40%)	14 (1.77%)	4 (0.96%)	19 (14.96%)	23 (4.22%)	2 (0.51%)	3 (4.35%)	5 (1.08%)
Moxifloxacin	71 (6.65%)	88 (37.45%)	159 (12.20%)	44 (7.64%)	29 (40.85%)	73 (11.28%)	9 (6.52%)	3 (23.08%)	12 (7.95%)	32 (4.62%)	39 (40.21%)	71 (8.99%)	33 (7.89%)	55 (43.31%)	88 (16.15%)	41 (10.38%)	20 (28.99%)	61 (13.15%)
Ofloxacin	72 (6.74%)	92 (39.15%)	164 (12.59%)	46 (7.99%)	29 (40.85%)	75 (11.59%)	9 (6.52%)	4 (30.77%)	13 (8.61%)	34 (4.91%)	43 (44.33%)	77 (9.75%)	32 (7.66%)	54 (42.52%)	86 (15.78%)	43 (10.89%)	20 (28.99%)	63 (13.58%)
Aminosalicylic acid	6 (0.56%)	10 (4.26%)	16 (1.23%)	4 (0.69%)	1 (1.41%)	5 (0.77%)	2 (1.45%)	2 (15.38%)	4 (2.65%)	3 (0.43%)	4 (4.12%)	7 (0.89%)	2 (0.48%)	5 (3.94%)	7 (1.28%)	3 (0.76%)	0 (0.00%)	3 (0.65%)
Rifabutin	51 (4.78%)	92 (39.15%)	143 (10.97%)	23 (3.99%)	29 (40.85%)	52 (8.04%)	9 (6.52%)	6 (46.15%)	15 (9.93%)	33 (4.76%)	47 (48.45%)	80 (10.13%)	17 (4.07%)	48 (37.80%)	65 (11.93)	15 (3.80%)	20 (28.99%)	35 (7.54%)
Rifampin	65 (6.09%)	113 (48.09%)	178 (13.66%)	27 (4.69%)	36 (50.70%)	63 (9.74%)	11 (7.97%)	8 (61.54%)	19 (12.58%)	37 (5.34%)	56 (57.73%)	93 (11.77%)	23 (5.50%)	64 (50.39%)	87 (15.96%)	21 (5.32%)	21 (30.43%)	42 (9.05%)
Streptomycin	129 (12.08%)	79 (33.62%)	208 (15.96%)	68 (11.81%)	26 (36.62%)	94 (14.53%)	17 (12.32%)	7 (53.85%)	24 (15.89%)	90 (12.99%)	40 (41.24%)	130 (16.46%)	47 (11.24%)	43 (33.86%)	90 (16.51%)	43 (10.89%)	15 (21.74%)	58 (12.50%)
Resistance to any first-line drug	193 (18.07%)	141 (60.00%)	334 (25.63%)	95 (16.49%)	44 (61.97%)	139 (21.48%)	23 (16.67%)	9 (69.23%)	32 (21.19%)	126 (18.18%)	64 (65.98%)	190 (24.05%)	71 (16.99%)	81 (63.78%)	152 (27.89%)	68 (17.22%)	31 (44.93%)	99 (21.34%)
Resistance to any drug	256 (23.97%)	153 (65.11%)	409 (31.39%)	136 (23.61%)	46 (64.79%)	182 (28.13%)	28 (20.29%)	9 (69.23%)	37 (24.50%)	155 (22.37%)	66 (68.04%)	221 (27.97%)	103 (24.64%)	89 (70.08%)	192 (35.23%)	106 (26.84%)	35 (50.72%)	141 (30.39%)
MDR	53 (4.96%)	99 (42.13%)	152 (11.67%)	21 (3.65%)	35 (49.30%)	56 (8.66%)	9 (6.52%)	8 (61.54%)	17 (11.26%)	30 (4.33%)	53 (54.64%)	83 (10.51%)	19 (4.55%)	56 (44.09%)	75 (13.76%)	16 (4.05%)	17 (24.64%)	33 (7.11%)
XDR	4 (0.37%)	22 (9.36%)	26 (2.00%)	1 (0.17%)	5 (7.04%)	6 (0.93%)	3 (2.17%)	0 (0.00%)	3 (1.99%)	1 (0.14%)	11 (11.34%)	12 (1.52%)	1 (0.24%)	13 (10.24%)	14 (2.57%)	0 (0.00%)	3 (4.35%)	3 (0.65%)

At the same time, Table 3 shows the drug resistance profile of initial treated TB and re-treated TB in different genders and age groups. In male groups, the drug resistance rates of amikacin, ethambutol, and kanamycin in re-treated TB were more than 10 times higher than those in initial treated TB. In female groups, the drug resistance rates of amikacin, ethambutol, kanamycin, rifabutin, and rifampin in re-treated TB were more than 10 times higher than those in initial treated TB. Similarly, in different age groups, the drug resistance rates of multiple drugs in re-treatment TB were more than 10 times higher than those in initial treated TB.

### Drug Resistance Profile in Different Regions

Details of resistance to any first-line drug, resistance to any drug, MDR-TB and XDR-TB in different regions are detailed in Table 4. The proportion of re-treated TB cases in Shanghai (11.38%, 88/773) was significantly lower than that in Jiangsu Province (21.43%, 63/294), Anhui Province (21.23%, 45/212), Zhejiang Province (15.51%, 29/187), and Jiangxi Province (21.97%, 29/132).

**Table 4:** The drug resistance profile in different regions.

**Drug**	**Shanghai (*****N*** **= 773)**			**Jiangsu Province (*****N*** **= 294)**			**Anhui Province (*****N*** **= 212)**			**Zhejiang Province (*****N*** **= 187)**		
	**Initial treated TB (*N* = 685)**	**Re-treated TB (*N* = 88)**	**Total TB**	**Initial treated TB (*N* = 231)**	**Re-treated TB (*N* = 63)**	**Total TB**	**Initial treated TB (*N* = 167)**	**Re-treated TB (*N* = 45)**	**Total TB**	**Initial treated TB (*N* = 158)**	**Re-treated TB (*N* = 29)**	**Total TB**
Amikacin	2 (0.29%)	5 (5.68%)	7 (0.91%)	0 (0.00%)	6 (9.52%)	6 (2.04%)	3 (1.80%)	6 (13.33%)	9 (4.25%)	1 (0.63%)	2 (6.90%)	3 (1.60%)
Cycloserine	9 (1.31%)	4 (4.55%)	13 (1.68%)	6 (2.60%)	5 (7.94%)	11 (3.74%)	4 (2.40%)	2 (4.44%)	6 (2.83%)	4 (2.53%)	5 (17.24%)	9 (4.81%)
Ethambutol	6 (0.88%)	9 (10.23%)	15 (1.94%)	2 (0.87%)	15 (23.81%)	17 (5.78%)	8 (4.79%)	14 (31.11%)	22 (10.38%)	1 (0.63%)	5 (17.24%)	6 (3.21%)
Ethionamide	3 (0.44%)	3 (3.41%)	6 (0.78%)	3 (1.30%)	6 (9.52%)	9 (3.06%)	3 (1.80%)	4 (8.89%)	7 (3.30%)	1 (0.63%)	2 (6.90%)	3 (1.60%)
Isoniazid	64 (9.34%)	30 (34.09%)	94 (12.16%)	29 (12.55%)	34 (53.97%)	63 (21.43%)	29 (17.37%)	27 (60.00%)	56 (26.42%)	27 (17.09%)	17 (58.62%)	44 (23.53%)
Kanamycin	4 (0.58%)	5 (5.68%)	9 (1.16%)	0 (0.00%)	8 (12.70%)	8 (2.72%)	4 (2.40%)	7 (15.56%)	11 (5.19%)	1 (0.63%)	3 (10.34%)	4 (2.14%)
Moxifloxacin	50 (7.30%)	24 (27.27%)	74 (9.57%)	18 (7.79%)	23 (36.51%)	41 (13.95%)	14 (8.38%)	20 (44.44%)	34 (16.04%)	10 (6.33%)	12 (41.38%)	22 (11.76%)
Ofloxacin	50 (7.30%)	24 (27.27%)	74 (9.57%)	18 (7.79%)	23 (36.51%)	41 (13.95%)	15 (8.98%)	20 (44.44%)	35 (16.51%)	9 (5.70%)	12 (41.38%)	21 (11.23%)
Aminosalicylic acid	4 (0.58%)	2 (2.27%)	6 (0.78%)	1 (0.43%)	4 (6.35%)	5 (1.70%)	1 (0.60%)	2 (4.44%)	3 (1.42%)	0 (0.00%)	2 (6.90%)	2 (1.07%)
Rifabutin	19 (2.77%)	17 (19.32%)	36 (4.66%)	14 (6.06%)	29 (46.03%)	43 (14.63%)	13 (7.78%)	27 (60.00%)	40 (18.87%)	9 (5.70%)	7 (24.14%)	16 (8.56%)
Rifampin	23 (3.36%)	23 (26.14%)	46 (5.95%)	17 (7.36%)	30 (47.62%)	47 (15.99%)	17 (10.18%)	31 (68.89%)	48 (22.64%)	11 (6.96%)	14 (48.28%)	25 (13.37%)
Streptomycin	62 (9.05%)	21 (23.86%)	83 (10.74%)	31 (13.42%)	22 (34.92%)	53 (18.03%)	33 (19.76%)	20 (44.44%)	53 (25.00%)	21 (13.29%)	11 (37.93%)	32 (17.11%)
Resistance to any first-line drug	94 (13.72%)	37 (42.05%)	131 (16.95%)	47 (20.35%)	37 (58.73%)	84 (28.57%)	44 (26.35%)	33 (73.33%)	77 (36.32%)	33 (20.89%)	17 (58.62%)	50 (26.74%)
Resistance to any drug	141 (20.58%)	45 (51.14%)	186 (24.06%)	61 (26.41%)	39 (61.90%)	100 (34.01%)	51 (30.54%)	34 (75.56%)	85 (40.09%)	42 (26.58%)	19 (65.52%)	61 (32.62%)
MDR	16 (2.34%)	21 (23.86%)	37 (4.79%)	14 (6.06%)	27 (42.86%)	41 (13.95%)	13 (7.78%)	26 (57.78%)	39 (18.40%)	10 (6.33%)	14 (48.28%)	24 (12.83%)
XDR	1 (0.15%)	4 (4.55%)	5 (0.65%)	0 (0.00%)	4 (6.35%)	4 (1.36%)	3 (1.80%)	6 (13.33%)	9 (4.25%)	0 (0.00%)	3 (10.34%)	3 (1.60%)
**Drug**	**Jiangxi Province (*****N*** **= 132)**			**Eastern regions (*****N*** **= 1,350)**			**Central regions (*****N*** **= 464)**			**Western regions (*****N*** **= 136)**		
	**Initial treated TB (*****N*** **= 103)**	**Re-treated TB (*****N*** **= 29)**	**Total TB**	**Initial treated TB (*****N*** **= 1,154)**	**Re-treated TB (*****N*** **= 196)**	**Total TB**	**Initial treated TB (*****N*** **= 377)**	**Re-treated TB (*****N*** **= 87)**	**Total TB**	**Initial treated TB (*****N*** **= 113)**	**Re-treated TB (*****N*** **= 23)**	**Total TB**
Amikacin	1 (0.97%)	4 (13.79%)	5 (3.79%)	3 (0.26%)	18 (9.18%)	21 (1.56%)	4 (1.06%)	11 (12.64%)	15 (3.23%)	2 (1.77%)	1 (4.35%)	3 (2.21%)
Cycloserine	3 (2.91%)	0 (0.00%)	3 (2.27%)	24 (2.08%)	15 (7.65%)	39 (2.89%)	9 (2.39%)	2 (2.30%)	11 (2.37%)	5 (4.42%)	0 (0.00%)	5 (3.68%)
Ethambutol	1 (0.97%)	14 (48.28%)	15 (11.36%)	12 (1.04%)	36 (18.37%)	48 (3.56%)	12 (3.18%)	34 (39.08%)	46 (9.91%)	2 (1.77%)	4 (17.39%)	6 (4.41%)
Ethionamide	0 (0.00%)	6 (20.69%)	6 (4.55%)	8 (0.69%)	13 (6.63%)	21 (1.56%)	5 (1.33%)	12 (13.79%)	17 (3.66%)	1 (0.88%)	1 (4.35%)	2 (1.47%)
Isoniazid	9 (8.74%)	18 (62.07%)	27 (20.45%)	133 (11.53%)	95 (48.47%)	228 (16.89%)	51 (13.53%)	54 (62.07%)	105 (22.63%)	14 (12.39%)	14 (60.87%)	28 (20.59%)
Kanamycin	1 (0.97%)	5 (17.24%)	6 (4.55%)	5 (0.43%)	21 (10.71%)	26 (1.93%)	6 (1.59%)	14 (16.09%)	20 (4.31%)	1 (0.88%)	1 (4.35%)	2 (1.47%)
Moxifloxacin	9 (8.74%)	14 (48.28%)	23 (17.42%)	81 (7.02%)	69 (35.20%)	150 (11.11%)	28 (7.43%)	39 (44.83%)	67 (14.44%)	6 (5.31%)	9 (39.13%)	15 (11.03%)
Ofloxacin	9 (8.74%)	15 (51.72%)	24 (18.18%)	81 (7.02%)	70 (35.71%)	151 (11.19%)	29 (7.69%)	41 (47.13%)	70 (15.09%)	8 (7.08%)	10 (43.48%)	18 (13.24%)
Aminosalicylic acid	0 (0.00%)	0 (0.00%)	0 (0.00%)	5 (0.43%)	9 (4.59%)	14 (1.04%)	3 (0.80%)	2 (2.30%)	5 (1.08%)	2 (1.77%)	0 (0.00%)	2 (1.47%)
Rifabutin	3 (2.91%)	17 (58.62%)	20 (15.15%)	48 (4.16%)	61 (31.12%)	109 (8.07%)	18 (4.77%)	50 (57.47%)	68 (14.66%)	8 (7.08%)	10 (43.48%)	18 (13.24%)
Rifampin	5 (4.85%)	17 (58.62%)	22 (16.67%)	58 (5.03%)	81 (41.33%)	139 (10.30%)	27 (7.16%)	56 (64.37%)	83 (17.89%)	7 (6.19%)	12 (52.17%)	19 (13.97%)
Streptomycin	11 (10.68%)	7 (24.14%)	18 (13.64%)	127 (11.01%)	64 (32.65%)	191 (14.15%)	57 (15.12%)	33 (37.93%)	90 (19.40%)	13 (11.50%)	8 (34.78%)	21 (15.44%)
Resistance to any first-line drug	14 (13.59%)	20 (68.97%)	34 (25.76%)	192(16.64%)	107 (54.59%)	299 (22.15%)	78 (20.69%)	63 (72.41%)	141 (30.39%)	18 (15.93%)	15 (65.22%)	33 (24.26%)
Resistance to any drug	24 (23.30%)	21 (72.41%)	45 (34.09%)	268 (23.22%)	119 (60.71%)	387 (28.67%)	101 (26.79%)	65 (74.71%)	166 (35.78%)	23 (20.35%)	15 (65.22%)	38 (27.94%)
MDR	4 (3.88%)	15 (51.72%)	19 (14.39%)	45 (3.90%)	72 (36.73%)	117 (8.67%)	22 (5.84%)	50 (57.47%)	72 (15.52%)	7 (6.19%)	12 (52.17%)	19 (13.97%)
XDR	0 (0.00%)	3 (10.34%)	3 (2.27%)	1 (0.09%)	15 (7.65%)	16 (1.19%)	3 (0.80%)	11 (12.64%)	14(3.02%)	1 (0.88%)	1 (4.35%)	2 (1.47%)

The drug resistance rates of most drugs in Shanghai are lower than those in other regions, regardless treatment history (Table 4). The percentages of MDR/XDR-TB in Shanghai (4.79 and 0.65%) were significantly lower than those in Jiangsu Province (13.95 and 1.36%), Zhejiang Province (12.83 and 1.60%), Jiangxi Province (14.39 and 2.27%) and Anhui Province (18.40 and 4.25%). The proportions of MDR-TB and XDR-TB were significantly higher in central regions (15.52 and 3.02%) than those in the two other regions (8.67 and 1.19% in eastern regions, 13.97 and 1.47% in western regions).

### MIC Determination and Distribution

**Figure 3:**
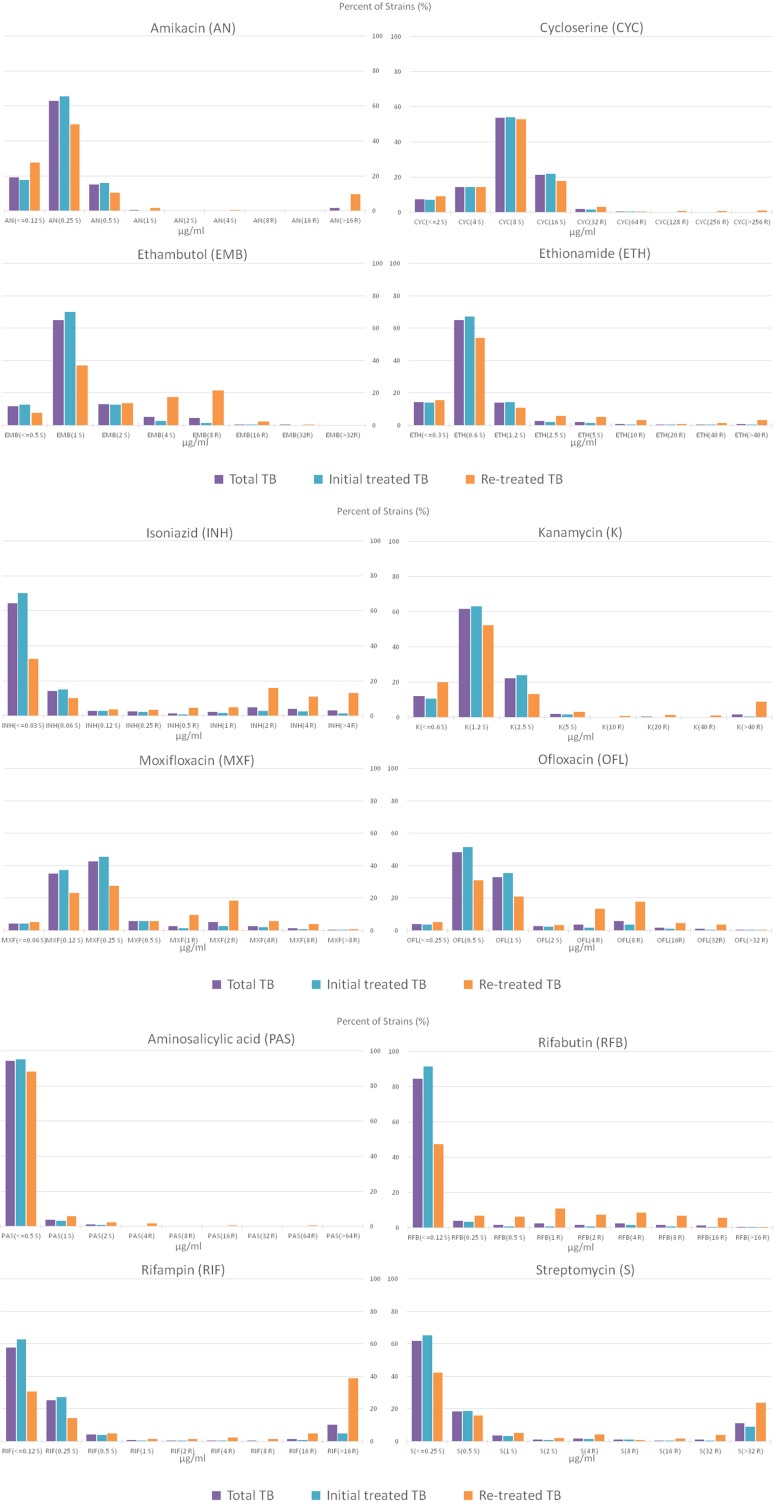
Percentage distribution plot of MICs of amikacin, cycloserine, ethionamide, ethambutol, isoniazid, kanamycin, moxifloxacin, ofloxacin, aminosalicylic acid, rifabutin, rifampin and streptomycin, against MTB isolates isolated from total TB, initial treated TB and re-treated TB. S and R after the MIC concentration represent sensitivity and resistance, respectively.

The overall MIC distributions for 12 drugs are shown in Figure 3. The majority of the amikacin-resistant isolates (36/39, 92.31%) had a MIC of >16 μg/mL. Meanwhile, most resistant isolates to kanamycin (68.75%, 33/48), rifampin (81.74%, 197/241) and streptomycin (72.19, 218/302) also showed high levels of resistance (a MIC of >40 μg/mL for kanamycin, a MIC of >16 μg/mL for rifampin and a MIC of >32 μg/mL for streptomycin).

Most cycloserine-resistant isolates (80.00%, 44/55) and ethambutol-resistant isolates (89.00%, 89/100) showed low levels of resistance (MICs of 32 and 64 μg/mL for cycloserine, a MIC of 8 μg/mL for ethambutol). Especially, no strain had a MIC of >32 μg/mL among ethambutol-resistant isolates. As shown in Figure 3, very few of the resistant isolates to moxifloxacin and ofloxacin show high levels of resistance, 2.16% (5/232) with a MIC of >8 μg/mL for moxifloxacin and 0.84% (2/239) with a MIC of >32 μg/mL for ofloxacin, respectively.

### Characteristics on the 33 Rifampicin-Resistant but Isoniazid-Sensitive Isolates

Out of the 33 rifampicin-resistant but isoniazid-sensitive isolates tested, 18 were initial treated TB and 15 were re-treated TB. Twenty-six cases were male and 7 cases were female. Among these cases, 2 were younger than 20 years old, 10 were 20–40 years old, 12 were 41–60 years old, and 9 were older than 60 years old.

Among these 33 strains, 30 had a MIC of >16 μg/mL and 3 had a MIC of 16 μg/mL for rifampicin.

## Discussion

In this study, we used Myco TB to investigate drug resistance characteristics of MTB isolates. The MIC Plate for testing is a 96-well microplate, which contains 12 antituberculous drugs with appropriate concentration dilutions and two positive controls. Myco TB is now used as the phenotypic susceptibility testing in large TB laboratories, and it has been demonstrated to have a high accuracy with >90% compared to Bactec MGIT 960 and the agar proportion method (Hall et al., 2012; Mpagama et al., 2013; Lee et al., 2014; Heysell et al., 2015; Mokrousov et al., 2016; Yu et al., 2016a). Myco TB has advantages due to its cost and more susceptibility information. In addition, Myco TB plate is fixed with the corresponding concentration of anti-tuberculosis drugs, which can be stored for 2 years at room temperature, thus avoiding the preparation and storage of reagents, and reducing the differences between laboratories.

Consistent with previous studies (Li et al., 2016; Liu et al., 2018), we found that the prevalence of TB is higher in male than in female. The male/female gender ratio in this study reached 2.01 (1,303/647), similar to the national estimated TB incidence in China for male (67.49%) and female (32.51%) patients (World Health Organization, 2018), which is lower than the gender ratio from the base-line surveillance of tuberculosis in 2007 (Zhao et al., 2012). At the same time, we found that the drug resistance rate of male to tuberculosis was significantly higher than that of female. This may be due to the different population distribution of the study population and different study periods. Besides gender, age is also associated with tuberculosis infection. We observed that more than half of the TB patients are between 20 and 50 years old, which may be related to their social relations and working conditions.

In this study, the incidences of MDR in initial treated TB cases were consistent with those found in global tuberculosis report 2018 and a national survey of drug-resistant tuberculosis in China (Zhao et al., 2012). And the incidences of MDR and XDR in re-treated TB cases were significantly higher than those in initial treated TB cases, indicating that previous treatment is a major risk factor for drug-resistant TB. It has been reported that the incidence of MDR in re-treated TB cases can be up to 10 times higher than that in initial treated TB cases (Pablos-Mendez et al., 1998). Misuse of anti-TB drugs, irregular treatment and poor patient compliance may result in drug resistance. Therefore, clinical emphasis on standardized anti-TB treatment can reduce the incidence of drug-resistant tuberculosis. Therefore, the implementation of standardized anti-tuberculosis treatment is still very important for the effective control of drug-resistant TB and MDR-TB, especially in supervising patients to complete treatment. We found that the incidence of MDR in re-treated patients in our study was significantly higher than that reported in global tuberculosis report 2018 (World Health Organization, 2018). It may because that SPH is a well-known hospital for tuberculosis treatment in China, and attract many patients with drug-resistant tuberculosis and poor anti-TB treatments in other hospitals before.

This study has shown that patients older than 60 years had significantly lower percentages of MDR/XDR-TB than in younger age groups which are comparable to other studies (Law et al., 2008; Rifat et al., 2014; Ullah et al., 2016). We supposed that the differences in age may be that young people are often busy working, studying and living under great pressure, while the lifestyle of the elderly is relatively simple. Moreover, our study shows that this difference is more significant in re-treated cases, and the drug resistance rate of re-treated patients older than 60 years is significantly lower than that of other age groups. This indicates that the imbalance of drug resistance among different age groups is mainly caused by treatment history.

Meanwhile, the proportion of re-treated cases in Shanghai was significantly lower than that in other regions, which suggests that the initial treated patients in Shanghai can get better treatment, leading to fewer relapses. The proportions of MDR-TB and XDR-TB in Shanghai were also significantly lower than that in other provinces, autonomous regions and municipalities in China. This should be due to Shanghai's developed economy, abundant medical resources and a relatively complete TB prevention and control system.

The second point we found in this study 208 isolates were resistant to isoniazid in the 241 RR-TB cases. Previously studies have demonstrated that about 90% RR-TB patients were also resistant to isoniazid (Yin et al., 2016). Therefore, rifampin susceptibility testing can be used to screen MDR. Meanwhile, 33 rifampicin-resistant but isoniazid-sensitive isolates showed high levels of resistance for rifampicin.

Rifabutin is a semi-synthetic antibiotic derived from rifampin S and is part of rifampin family together with rifampin (Marsili et al., 1981). Rifabutin has been suggested as a reasonable alternative to treat MDR and XDR associated with particular *rpoB* mutations although cross-resistance to rifabutin and rifampin is common in previous studies (Sirgel et al., 2013), and in our study, 80.08% (193/241) isolates from RR-TB patients were resistant to rifabutin. At the same time, our study also shows that ofloxacin and moxifloxacin, amikacin, and kanamycin also have high cross-resistances, which had been reported in other studies (Alangaden et al., 1998; Kam et al., 2006; Sowajassatakul et al., 2014; Yu et al., 2016b).

In addition to ethambutol, the other three first-line drugs (isoniazid, rifampin and streptomycin) have very high drug resistance rates, especially isoniazid (361/1,950, 18.51%). Nearly a quarter of cases (473/1,950, 24.26%) are resistant to at least one first-line drug. Therefore, more second-line drugs must be used in anti-TB treatments, with more side effects and higher costs (World Health Organization, 2018). Among 208 MDR isolates, only 9.62 and 6.73% isolates were resistant to cycloserine and aminosalicylic acid, respectively. Cycloserine and aminosalicylic acid can be in the treatment of MDR-TB, but treatment history, adverse effects, and cost should be considered (World Health Organization, 2010).

Finally, the distribution of MIC showed that most of the amikacin-, kanamycin-, rifampin-, and streptomycin-resistant isolates showed high levels of resistance, while most of the cycloserine-, ethambutol-, moxifloxacin-, and ofloxacin-resistant isolates showed low levels of resistance.

Our study has several limitations. Firstly, although the specimens cover 29 provinces, autonomous regions and municipalities, all isolates were collected from only one hospital in Shanghai. Secondly, the DST panel does not include all anti-TB drugs, including capreomycin, linezolid, levofloxacin, other three commonly used second-line antituberculous. Thirdly, due to the retrospective study design, we did not have contacted with the patients, and some important data are not recorded, such as detailed drug regimens, side effects, any genetic background and molecular typing data, etc.

In conclusion, this study has updated drug resistance characteristics of MTB isolates to four first-line and eight second-line antituberculous drugs from tuberculosis patients in China. Our study found higher prevalence of MDR and XDR among re-treated cases than those among initial treated TB cases and the drug resistance rate of tuberculosis varied with age, sex and region, indicating that standardized anti-tuberculosis treatment is needed to reduce the incidence of drug-resistant tuberculosis and the recurrence of tuberculosis.

## Data Availability Statement

The datasets generated for this study are available on request to the corresponding author.

## Ethics Statement

This study was approved by the Ethics Committee of the Shanghai Pulmonary Hospital (SPH), affiliated to Tongji University, P.R. China. The patients enrolled in this study were all from SPH, and written informed consents were obtained from each of them.

## Author Contributions

FY designed the study. XW wrote the manuscript. BW and FY modified the manuscript. XW, GT, HL, YL, and JY did the statistics. JY, BW, RG, and YG did laboratory examination.

### Conflict of Interest

The authors declare that the research was conducted in the absence of any commercial or financial relationships that could be construed as a potential conflict of interest.
